# Glioma: bridging the tumor microenvironment, patient immune profiles and novel personalized immunotherapy

**DOI:** 10.3389/fimmu.2023.1299064

**Published:** 2024-01-11

**Authors:** Tatiana A. Mishchenko, Victoria D. Turubanova, Ekaterina N. Gorshkova, Olga Krysko, Maria V. Vedunova, Dmitri V. Krysko

**Affiliations:** ^1^Institute of Biology and Biomedicine, National Research Lobachevsky State University of Nizhny Novgorod, Nizhny Novgorod, Russia; ^2^Neuroscience Research Institute, National Research Lobachevsky State University of Nizhny Novgorod, Nizhny Novgorod, Russia; ^3^Cell Death Investigation and Therapy Laboratory, Anatomy and Embryology Unit, Department of Human Structure and Repair, Faculty of Medicine and Health Sciences, Ghent University, Ghent, Belgium; ^4^Faculty of Biology and Biotechnologies, National Research University Higher School of Economics, Moscow, Russia; ^5^Department of Pathophysiology, Sechenov First Moscow State Medical University (Sechenov University), Moscow, Russia; ^6^Cancer Research Institute Ghent, Ghent, Belgium

**Keywords:** glioma, cancer immunotherapy, immunogenic cell death, ICD, tumor microenvironment, Th17, anti-tumor vaccination, tumor neoantigens

## Abstract

Glioma is the most common primary brain tumor, characterized by a consistently high patient mortality rate and a dismal prognosis affecting both survival and quality of life. Substantial evidence underscores the vital role of the immune system in eradicating tumors effectively and preventing metastasis, underscoring the importance of cancer immunotherapy which could potentially address the challenges in glioma therapy. Although glioma immunotherapies have shown promise in preclinical and early-phase clinical trials, they face specific limitations and challenges that have hindered their success in further phase III trials. Resistance to therapy has been a major challenge across many experimental approaches, and as of now, no immunotherapies have been approved. In addition, there are several other limitations facing glioma immunotherapy in clinical trials, such as high intra- and inter-tumoral heterogeneity, an inherently immunosuppressive microenvironment, the unique tissue-specific interactions between the central nervous system and the peripheral immune system, the existence of the blood-brain barrier, which is a physical barrier to drug delivery, and the immunosuppressive effects of standard therapy. Therefore, in this review, we delve into several challenges that need to be addressed to achieve boosted immunotherapy against gliomas. First, we discuss the hurdles posed by the glioma microenvironment, particularly its primary cellular inhabitants, in particular tumor-associated microglia and macrophages (TAMs), and myeloid cells, which represent a significant barrier to effective immunotherapy. Here we emphasize the impact of inducing immunogenic cell death (ICD) on the migration of Th17 cells into the tumor microenvironment, converting it into an immunologically “hot” environment and enhancing the effectiveness of ongoing immunotherapy. Next, we address the challenge associated with the accurate identification and characterization of the primary immune profiles of gliomas, and their implications for patient prognosis, which can facilitate the selection of personalized treatment regimens and predict the patient’s response to immunotherapy. Finally, we explore a prospective approach to developing highly personalized vaccination strategies against gliomas, based on the search for patient-specific neoantigens. All the pertinent challenges discussed in this review will serve as a compass for future developments in immunotherapeutic strategies against gliomas, paving the way for upcoming preclinical and clinical research endeavors.

## 1 Introduction

Glioma, the most common and deadliest malignant primary brain tumor in adults, poses a formidable challenge. However, despite the constant updating and improvement of neurosurgical techniques and sophisticated immunotherapeutic regimens (*e.g.*, chimeric antigen receptor T-cell and NK-cell therapies, immune checkpoint inhibitors, gene-mediated cytotoxic immunotherapy and oncolytic viruses), the survival rate of patients with glioma remains very low and prognosis very poor in terms of survival and quality of life (1–5).

Gliomas usually originate from glial cells and affect both the brain and spinal cord. The 2021 WHO Classification of Tumors of the CNS, 5^th^ edition (WHO CNS 5) recognizes the following four families of gliomas: 1) adult-type diffuse gliomas; 2) pediatric-type diffuse low-grade gliomas; 3) pediatric-type diffuse high-grade gliomas; and 4) circumscribed astrocytic gliomas (6, 7). The current treatment of glioma is ineffective, prolonging the patient’s life by only 2.5 years. Therefore, there is an active search for new therapeutic strategies that could give hope to thousands of patients. According to the aforementioned WHO classification of CNS tumors (WHO CNS 5), grading malignancy and choosing a therapeutic strategy emphasize the molecular markers (6, 8), sometimes even more than the histological features (9). This approach can yield powerful prognostic information (10, 11). In addition, several important molecular alterations for characterizing the genetic profile of gliomas have been identified such as isocitrate dehydrogenase 1 and 2 (IDH1, IDH2), telomerase reverse transcriptase (TERT, including promoter region), O-6-methylguanine-DNA methyltransferase (MGMT) and alpha-thalassemia/mental retardation, X-linked, B-Raf proto-oncogene serine/threonine kinase, tumor protein p53, epidermal growth factor receptor amplification or mutation, cyclin-dependent kinase inhibitor 2A or 2B, codeletion of chromosome arms 1p and 19q, combined gain of chromosome 7 and loss of chromosome 10. The most notable molecular subtypes of glioma, according to the WHO Classification of Tumors of the CNS, 4th edition, are IDH mutations and the 1p/19q deletion (12). Moreover, the molecular features have been found to be associated with distinct immune landscapes and prognosis, suggesting a link between molecular and immune subtypes in gliomas (10, 13–18).

Modern therapeutic approaches to the treatment of gliomas rely on clinical presentation, tumor grade, tumor size, and tumor location. However, surgery, radiotherapy (RT) and systemic therapy, including chemotherapy and targeted therapy, remain the main approaches. Combinations of these methods are usually applied to contain the progression of the disease (**Table 1**).

Table 1Main features and therapeutic approaches for the treatment of gliomas according to the 5th edition (2021) update to the WHO classification of CNS tumors.

**Table:** 

Adult-type diffuse gliomas							
Type	Astrocytoma, IDH-mutant			Oligodendroglioma, IDH-mutant, and 1p/19q-codeleted		Glioblastoma, IDH-wildtype	Refs.
Molecular marker	IDH mutation			IDH mutation 1p19q codeletion		IDH-wildtype	(6, 19–22)
Grade	2	3	4	2	3	4	(6, 8)
Features	• low mitotic activity • CDKN2A/B (-)	• anaplasia • significant mitotic activity • CDKN2A/B(-)	• necrosis and/or • microvascular proliferation • CDKN2A/B(+)	• for grade 3: • increased cellular density • increased mitotic activity • microvascular proliferation and necrosis nuclear anaplasia (common)		• microvascular proliferation • necrosis	(6, 8, 22, 23)
						• EGFR gene amplification • TERT promoter mutation • EGFR gene amplification and/or a +7/–10 cytogenetic signature • MGMT promoter methylation • CDKN2A deletions	(6, 8, 23, 24)
Conventional treatment	• biopsy • surgical resection if feasible • radiotherapy at the time of recurrence or progression.	• surgical resection • concurrent chemoradiotherapy (Stupp protocol)					(6, 21, 25–27)
						antiangiogenesis (e.g. bevacizumab) and immunotherapy

**Table:** 

Pediatric-type diffuse low-grade gliomas					
Type	Diffuse astrocytoma MYB or MYBL1 altered	Angiocentric glioma	Polymorphous low-grade neuroepithelial tumor of the young	Diffuse low-grade glioma, MAPK pathway-altered	Refs.
Molecular marker	MYB or MYBL1 gene mutations	*MYB*	BRAF, FGFR family	FGFR1, BRAF, MYB/MYBL1 arrangement	(6, 28–32)
Grade	not applicable				
Pediatric-type diffuse low-grade gliomas					
	Diffuse astrocytoma MYB or MYBL1 altered	Angiocentric glioma	Polymorphous low-grade neuroepithelial tumor of the young	Diffuse low-grade glioma, MAPK pathway-altered	Refs.
Features	• non-specific and low-grade histopathologic features • rare or absent mitotic activity	• monomorphic population of elongated spindle-shaped bipolar cells with a strikingly perivascular orientation • strong predilection for perivascular spread • subpial growth along the surface of the cortex	• oligodendroglioma-like cellular elements • calcifications • strong CD34 immunopositivity	• broad spectrum of histological features, including astrocytic, oligodendroglial, or mixed morphology	(6)
Conventional treatment	Resection with good prognosis				(6, 33–35)

**Table:** 

Pediatric-type diffuse high-grade gliomas								
Type	Diffuse midline glioma, H3 K27-altered		Diffuse hemispheric glioma H3 G34 mutant		Diffuse pediatric-type high-grade gliomas, H3-wildtype and IDH-wildtype	Infant-type hemispheric glioma		Refs.
Molecular marker	• K27M mutations in the histone H3 gene H3F3A and related HIST1H3B genes • H3 K27, TP53, ACVR1, PDGFRA, EGFR, EZHIP		H3 G34, *TP53*, *ATRX*		IDH-wildtype, H3-wildtype, PDGFRA, MYCN, EGFR (methylome) Molecular subtypes: • RTK2 • RTK1 • MYCN	NTRK family, ALK, ROS, MET Molecular subtypes: • *NTRK*-altered • *ROS1*-altered • *ALK*-altered • *MET*-altered		(6, 36)
Grade	4							(6)
Features	Similar to astrocytic tumors, biopsies are not informative		• astrocytic cells with glioblastoma-like features • microvascular proliferation and necrosis • an embryonal-like appearance with smaller cells and hyperchromatic nuclei		• glioblastoma-like features with high cellularity • brisk mitotic activity • microvascular proliferation and necrosis	• astrocytic cells arranged in fascicles or sheets with mild to moderate pleomorphism • palisading necrosis • mitotic activity • endothelial proliferation		(6, 37–41)
Conventional treatment	Have no effective treatment, inoperable. Radiation therapy remains the only standard of care. (GD2-CAR T cell therapy)		Surgical resection with adjuvant radiotherapy and chemotherapy			Targeted therapy		(6, 40, 42, 43)
Circumscribed astrocytic gliomas								
Type	Pilocytic astrocytoma	High-grade astrocytoma with piloid features	Pleomorphic xanthoastrocytoma		Subependymal giant cell astrocytoma	Chordoid glioma	Astroblastoma, MN1-altered	Refs.
Molecular marker	• KIAA1549-BRAF, BRAF, NF1 IDH mutations (–) • TP53 mutations (–)	BRAF, NF1, ATRX, CDKN2A/B (methylome)	BRAF, CDKN2A/B		TSC1, TSC2	PRKCA	MN1	(6, 12, 44–47)
Grade	1	4	2, 3		1	2	Not applicable (varies)	(6)
Features	Heterogeneous • elongated hair-like projections from the neoplastic cells • eosinophilic Rosenthal fibers	Varies	Grade 3: necrosis and vascular proliferation		• arise from a subependymal nodule present in the ventricular wall in a patient with tuberous sclerosis • cysts and calcification	• clusters or cords of oval epithelioid cells embedded within a mucinous stroma with prominent lymphoplasmacytic infiltration and the presence of Russell bodies	• well-demarcated masses with areas of cystic degeneration and necrosis giving it a bubbly appearance	(6, 12, 45, 48, 49)
Conventional treatment	Surgical resection with good prognosis	Maximal safe surgical resection with concurrent chemoradiotherapy (e.g., TMZ)	Prognosis is good following surgical excision	Surgical excision, local recurrence and malignant transformation is common, neither radiotherapy nor chemotherapy has a significant effect	Surgery is often curative; oral mTOR inhibitors (e.g., everolimus or sirolimus)	Surgical resection, chemotherapy and/or radiotherapy is ineffective	Surgical resection with adjuvant radiation therapy and chemotherapy for high-grade lesions	(6, 49–55)

One of the initial steps in the treatment of gliomas is surgical resection, which removes the whole tumor and provides a biopsy for histological analysis and tumor genotyping (11, 56). However, there are limitations to the complete removal of gliomas by surgery due to their invasiveness in the surrounding tissues, and the peculiarities of their microenvironment, which lead to frequent relapses. To increase resection efficiency and control damage to healthy tissues, additional methods are used, such as fluorescence-guided resection with 5-aminolevulinic acid (5-ALA) (57–60), and motor and speech mapping through intraoperative cortical electrodes (61–63), along with intraoperative magnetic resonance imaging (MRI) (64, 65). In addition, to avoid subsequent malignant progression, surgical resection is often followed by RT alone or in combination with chemotherapy, depending on the tumor type (66–68). The choice of RT regimen (time, dosage, schedule) is based on the diagnosis and prognostic factors, including age, degree of resection, and Karnofsky score (69, 70). The Stupp protocol, named after Swiss oncologist Roger Stupp, has become the standard of care for the treatment of glioblastoma since its publication in 2005 and has led to significant prolongation of survival (**Box 1**). Modern methods of focused radiation therapy, such as intensity-modulated or image-guided radiation therapy and stereotactic radiation therapy, can improve the targeted delivery of RT for better protection of surrounding tissues (71, 72).

Box 1 **Stupp protocol of care for the treatment of glioblastoma.**

According to the original study, the Stupp protocol comprises radiotherapy (total 60 Gy; 2 Gy per day; Monday to Friday) over 6 weeks. Continuous daily TMZ at 75 mg/m^2^ of body-surface area per day, 7 days per week from the first to the last day of radiotherapy, followed by 6 cycles of adjuvant TMZ (150 to 200 mg/m^2^ for 5 days during each 28-day cycle) is also a part of the treatment protocol (69).

The use of chemotherapy in patients with glioma plays an important role in preventing postoperative recurrence. Modern glioma therapy practices use several different chemotherapeutic agents in combination with other treatment modalities to improve therapy efficacy (73). Although many drugs have been developed, the Food and Drug Administration (FDA) has approved only a few for the treatment of glioma, of which alkylating agents such as temozolomide (TMZ) are widely used. TMZ can methylate DNA, which most often occurs at the N-7 or O-6 position of guanine residues, inducing cell cycle arrest and apoptosis of cancer cells (73–77). MGMT promoter methylation is of the greatest clinical importance for predicting responses to TMZ (78, 79). However, TMZ toxicity and drug resistance limit its effectiveness, highlighting the importance of exploring and developing new treatment approaches (80–82). Another treatment option is chemotherapy with “second-line” nitrosourea-based drugs, including nimustine, carmustine, lomustine and ranimustine (83). Unfortunately, their action mechanisms, effectiveness, and side effects are comparable to those of TMZ and other alkylating agents, but they can be used to overcome TMZ resistance (77, 84, 85).

Another chemotherapeutic approach for glioma is platinum-based cancer therapy. Cisplatin, carboplatin and oxaliplatin have been widely used to treat brain tumors and other cancers (86). These drugs induce various cellular responses, such as cell cycle arrest, transcription inhibition, DNA repair, apoptosis (87, 88) and ICD (89, 90). The main limitations of these drugs are their instability, impossibility of oral administration, and poor permeability through the blood-brain barrier (BBB) (73). In addition, doxorubicin, vincristine and topotecan are used mainly in combination regimens (91–93).

Effective chemotherapeutic drugs with reduced side effects could be produced by using alternative drug delivery systems such as liposome encapsulation, peptide-protein conjugates, and polymeric micelles and nanoparticles. However, little success in delivery and side effects reduction have been achieved (73). Thus, there are currently not so many effective treatment options for glioma therapy, and the search for new approaches is a pressing issue.

New therapies based on molecular profiling, new small molecules, and novel immunotherapeutic approaches may improve the survival and quality of life of patients with gliomas. Now, there are high hopes for immunotherapy, as immunotherapeutic strategies have been repeatedly proven to be effective and safe and are likely to be increasingly used in the future. Several immunotherapeutic strategies are currently being applied or adapted in clinical trials for the treatment of gliomas. These strategies include the use of immune checkpoint inhibitors (94–96) (e.g., ClinicalTrials.gov: NCT04267146, NCT03557359, NCT03925246), chimeric antigen receptor (CAR) T cell therapy (97–99) (e.g., ClinicalTrials.gov: NCT06018363, NCT02208362, NCT01454596), oncolytic viruses (100, 101) (e.g., ClinicalTrials.gov: NCT06126744, NCT02062827, NCT00528684), gene-mediated cytotoxic immunotherapy (102) (e.g., ClinicalTrials.gov: NCT00751270, NCT03576612, NCT00589875) and therapeutic anti-tumor vaccination (103, 104) (e.g., ClinicalTrials.gov: NCT05283109, NCT03665545, NCT02507583). While all of these strategies hold promise as potential breakthroughs in glioma treatment, their efficacy and safety must be rigorously evaluated. Well-designed clinical trials are essential to address the challenges posed by the immunosuppressive tumor microenvironment, antigen heterogeneity, and antigen escape.

In this review, we first highlight the challenges presented by the glioma microenvironment and immune profiles of glioma, which pose a significant obstacle for effective immunotherapy, and highlight the impact of Th17 cells on the efficacy of glioma immunotherapy. Next, we delve into the primary immune profiles associated with gliomas and their implications for disease prognosis in patients and discuss a prospect approach to developing the most personalized vaccination strategy against glioma based on patient-specific neoantigens.

## 2 Bottleneck in glioma immunotherapy: the glioma microenvironment

**Figure 1:**
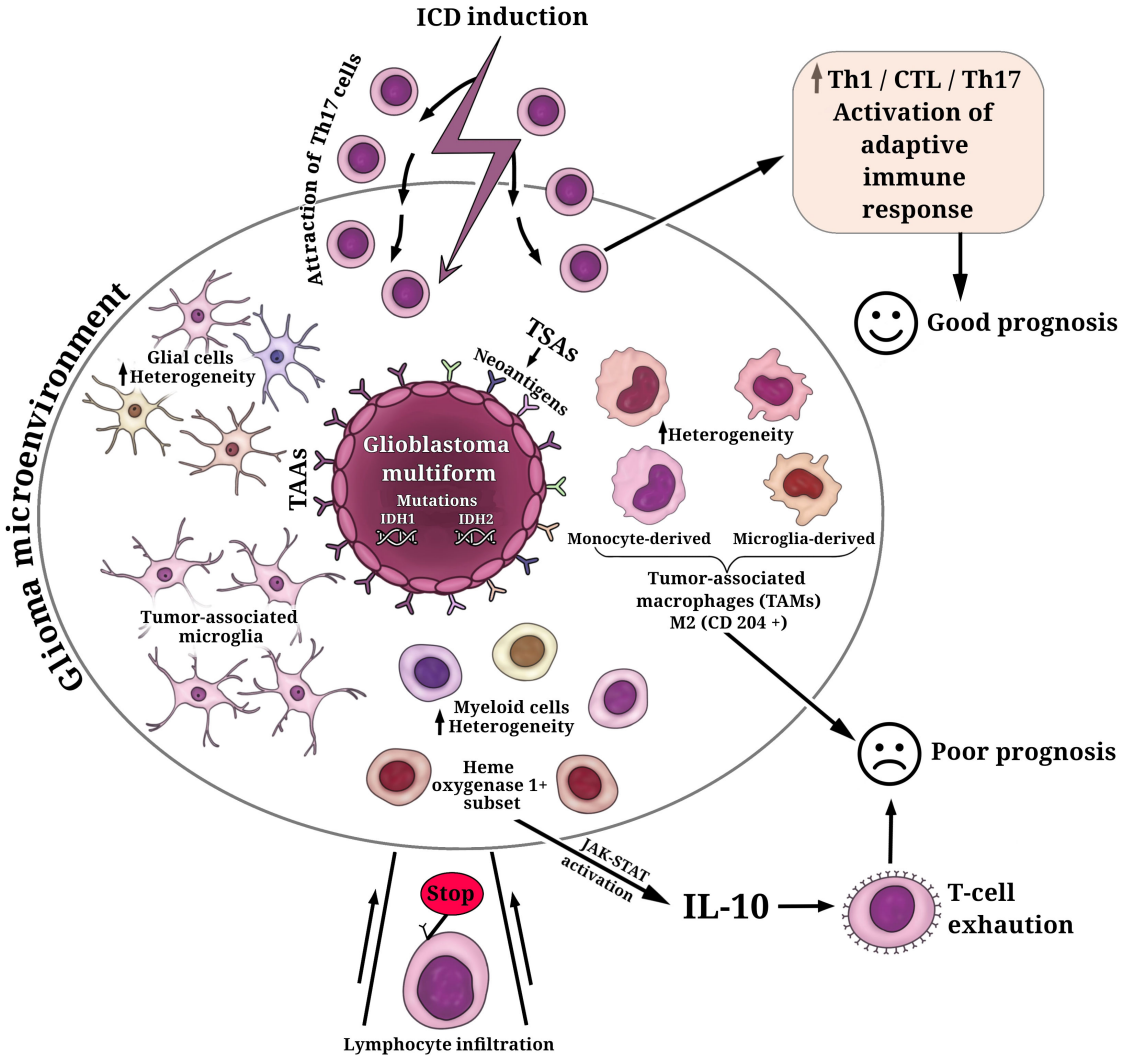
Key cell populations within the glioma microenvironment. The fundamental characteristic defining glioblastoma multiforme (GBM) is the presence of mutations in isocitrate dehydrogenases (IDH1, IDH2), which play a decisive role in determining tumor aggressiveness and treatment outcomes. GBM cells exhibit a repertoire of tumor-associated antigens (TAAs) and tumor-specific antigens (TSAs). Among these TSAs, personalized neoantigens resulting from patient-specific tumor mutations have gained significant interest in the development of personalized immunotherapeutic strategies. The cellular composition of the glioma microenvironment displays considerable variability, contributing to the inhibition of T cell infiltration and activation. The predominant population of infiltrating cells in the glioma microenvironment comprises robust tumor-associated microglia and macrophages (TAMs) (108). TAMs consist of microglia- or monocyte-derived populations, each exhibiting additional heterogeneity, including subsets with distinctive lipid and hypoxic signatures (109). Elevated levels of M2-like TAMs (CD204^+^ cells) have been associated with a more resistant, pro-tumorigenic microenvironment and a poorer prognosis for patients (110). Additionally, the glioma microenvironment houses glial and myeloid cell populations characterized by substantial heterogeneity (111). A subset of heme oxygenase 1^+^ myeloid cells, responsible for IL-10 release, triggers dysfunctional T cell transformation through activation of the JAK-STAT pathway—a major contributor to T-cell exhaustion and unfavorable prognoses (112). The induction of immunogenic cell death (ICD) in glioma cells can transition the immunological environment from ‘cold’ to ‘hot’ by attracting Th17 cells, thus activating an adaptive immune response (113–115). This transition is pivotal for enhancing the efficacy of ongoing glioma immunotherapy and fostering a positive prognosis for patients.

Glioblastoma multiforme (GBM, WHO grade 4) typically has a relatively low mutation load and immunologically ‘cold’ tumor microenvironment (TME) (105–107) that abrogates T-cell infiltration and activation (**Figure 1**). This is the current great challenge in immunotherapy. Importantly, the components of TME in GBM resemble those in other tumors, but they also include some unique brain tissue-resident cell types. To complicate matters, the cellular composition of the TME exhibits substantial variability among patients with GBM. More than 30% of infiltrating cells in the TME are robust tumor-associated microglia and macrophages (TAMs) (106, 108, 116). At the same time, glial and myeloid cell populations in the TME of GBM are highly heterogeneous, which can affect the efficacy of (immuno)therapeutic regimens and complicate the prediction of response to (immuno)therapy in individual patients (111) (**Figure 1**).

It has been reported that primary tumors of patients with GBM, which have increased levels of M2-like TAMs (CD204^+^ cells), were responsible for a more-resistant pro-tumorigenic microenvironment and thus were associated with GBM aggressiveness, treatment resistance, and poor prognosis (110) (**Figure 1**). It is of interest that another study also demonstrated a significant upregulation of M2 macrophages and their association with poor prognosis in patients with low-grade gliomas (117). However, recent studies have provided a breakthrough in characterizing and understanding the TME and the individual immune components, including the specific interactions between myeloid cells and glioma cells or myeloid cells and tumor-infiltrating lymphocytes. This progress renders the simplistic paradigm of M1/M2 phenotypes of TAMs no longer a viable option. In this regard, a recent study demonstrates substantial cellular and functional heterogeneity of myeloid cells in TME associated not only with different cell distributions in the tumor core and tumor periphery but also with sex-specific alterations in the responses of myeloid cells to gliomas (118). The study reveals significant upregulation of genes of the MHC II complex in microglia and monocytes/macrophages populations in male mice compared to female mice (118). High heterogeneity within the GBM myeloid compartment was uncovered through scRNA-seq and simultaneous epitope and transcriptome measurements in single cells (CITE-seq analysis) of the glioblastoma immune landscape in mouse tumors and in patients with newly diagnosed or recurrent disease (109). The authors revealed that microglia- or monocyte-derived populations of TAMs exhibit additional heterogeneity, including subsets with conserved lipid and hypoxic signatures. The dominance of microglia-derived TAMs was established in newly diagnosed tumors and suggests particular prognostic significance. In contrast, in recurrent tumors, the profile shifts to monocyte-derived TAMs, especially in hypoxic tumor environments (109). Equally interesting is the discovery of similarities between the human and mouse GBM immune compartments, highlighting the value of the GL261 experimental glioma as useful preclinical mouse model (109).

Of interest that scRNAseq and imaging mass cytometry-based single-cell profiling have provided evidence that hypoxic active regions in the tumor triggers cell-cycle arrest, particular an S-phase arrest (119) This contributes to the accumulation of genomic instabilities, facilitating microevolution toward resilience in GBM. Furthermore, both reactive-immune and hypoxia areas revealed a significant enrichment of TAMs and exhausted T cells suggesting a local enhanced immunosuppression (119). It is noteworthy that a recent study has shown the presence of a subset of heme oxygenase 1^+^ myeloid cells in the GBM microenvironment (112). These cells are responsible for releasing IL-10, which leads to dysfunctional T cell transformation *via* activation of the JAK-STAT pathway, which is a major driving force behind T-cell exhaustion (**Figure 1**).

Importantly, recent scRNAseq analysis of human glioma specimens (i.e., low-grade gliomas, newly diagnosed GBMs, and recurrent GBMs) has allowed the identification of nine distinct myeloid cell subtypes with unique gene expression patterns (MC1–MC9). Among these, five subtypes (MC2–MC5, and MC7) have shown the potential to independently serve as prognostic markers for patient survival, on par with established markers such as IDH mutation and MGMT gene methylation status (120). Activated (MC7) or homeostatic (MC2) microglia were associated with improved overall survival, whereas macrophage (MC4) and suppressive bone marrow-derived macrophage (MC3, MC5) signatures were associated with worse survival (120). The data also demonstrate high heterogeneity in types and quantities of immune cells in different regions of the same tumor specimen. Using an experimental mouse glioma model, the authors further demonstrated the critical role of gene expression of a small calcium-binding protein S100A4, which is also considered as a damage-associated molecular pattern (DAMPs) molecule (**Box 2**). This protein plays a role in GBM-associated T cells and pro-tumorigenic myeloid cells, promoting immunosuppression and glioma growth. The improved survival rate of S100a4^−/−^ glioma-bearing mice was associated with the generation of anti-tumor immunity, accompanied by increased phagocytic activity of CD11b^+^ glioma-associated myeloid cells and enhanced activity of CD4^+^ T cells, stimulating T cell proliferation and IFNγ secretion (120).

Box 2 **Immunogenic cell death and its role in anti-cancer therapy at a glance.**

Immunogenic cell death (ICD) is an umbrella term for several regulated cell death modalities characterized by the release of a pool of signaling immune-modulatory molecules, also known as damage-associated molecular patterns (DAMPs), to activate both innate and adaptive immunity and induce long-term immunological memory. Several DAMPs have already been well described, including small molecular weight molecules (e.g., ATP, F-actin) (121, 122), proteins (e.g., high-mobility group **Box 1** (HMGB1) (123), heat shock proteins HSP70 and HSP90, сalreticulin, type I interferon (IFNs)) (124, 125), nucleic acids (e.g., mRNA and genomic DNA) (126, 127), and lipids (e.g., cardiolipin) (128, 129)). This list is still growing. DAMPs emission can be triggered by a variety of stress factors, such as temperature exposure (130, 131), physico-chemical influences (132–134), viral load (135, 136), enzymatic processes (137), and cytotoxic effects mediated by T cells or activated NK lymphocyte (138). But of particular interest is the possibility of ICD induction and DAMPs release in ongoing anticancer therapy.

During induction of cell death in cancer cells, the DAMPs emitted act as “find me” and/or “eat me” signals to phagocytes. These are necessary adjuvant signals to attract antigen-presenting cells (i.e., DCs). Another important feature of cancer cells undergoing ICD is their antigenicity, which is driven by tumor-associated antigens (TAAs) and tumor-specific antigens (TSAs). This is followed by their presentation on major histocompatibility complex class I (MHC I) molecules to CD8^+^ T cells of the adaptive immune system. This is a major driving force of the effective control of tumor growth and long-term anti-cancer immunity (139, 140).

In 2005, the group led by Guido Kroemer first described a process of immunogenic apoptosis in doxorubicin-induced cancer cells. When cancer cells undergoing this form of cell death are used as a prophylactic vaccine, they significantly reduce tumor growth and activate long-term immunological memory in immunocompetent mice (141). Since then, the immunogenic properties of other types of cell death modalities have been identified, including necroptosis (142, 143), ferroptosis (144) and paraptosis (145), which can be induced by different anti-cancer treatment methods (e.g., chemotherapy, photodynamic and photothermal therapy, radiotherapy and their combinations) (139, 146). Remarkably, anti-cancer treatment in specific regimens can frequently trigger mixed types of cell death (147–149), which is considered useful for dealing with tumor cell death resistance and preventing cancer recurrence.

On the other hand, cancer cell death might not follow the immunogenic pathway when exposed to high doses of chemical agents and/or extreme physico-chemical stresses (e.g., heat, osmotic shock, mechanical, freeze-thawing). In this case, cancer cell death will follow the path of accidental necrosis, which leads to an inflammatory response rather than an immune response (150). Thus, the important prerequisite for induction of ICD in cancer cells is a delicate exposure to the death-inducing stimuli that cause induction of regulated death without sudden rupture of the plasma membrane leading to the release of the entire cellular content (151).

ICD can be determined by a combination of *in vitro* methods, including identification of cell death type by inhibitor analysis, identification of DAMPs profile emitted from dying cancer cells, analysis of phagocytic activity, and phenotypic status of antigen-presenting cells (i.e., dendritic cells) in the presence of dead/dying cancer cells (152). Importantly, the immunogenicity of cancer cell death should be proven also *in vivo*. The “gold standard” protocol for evaluating ICD in oncological mouse models has been summarized by Juliette Humeau et al. in 2019 (153). The protocol is based on prophylactic vaccination with treated dying/dead tumor cells of syngeneic immunocompetent animals followed by rechallenge with living entities of the same type of cancer. The absence or significant decrease in tumor growth at the challenged site indicates the immunogenicity of the dying/dead cancer cells.

To sum up, deeper investigations into the precise composition of the glioma TME, especially the intricacies of cell-to-cell interactions and their expression patterns, have the potential to facilitate the mitigation of resistance to immunotherapy. This represents an intriguing direction for future research.

## 3 An emerging role of Th17 cells in glioma microenvironment

In parallel with the immunologically ‘cold’ TME and its high heterogeneity, the peculiarities of T-cell function and the immune environment may lead to controversial effects on tumor progression and patient survival. Specifically, the role of Th17 cells, a subpopulation of effector CD4^+^ helper T cells that produce IL-17A, in the effectiveness of glioma immunotherapy continues to be a subject of extensive discussion. It has been demonstrated that Th17 cells, a subset of IL-17^+^ cells, exhibit a distinct correlation with improved cancer patient survival compared to the overall IL-17^+^ cell population. On the one hand, IL-17 primarily promotes tumorigenesis (113, 154, 155); but on the other, the subpopulation of IL-17 producing Th17 cells have a tumor-suppressing effect. The high level of Th17 cells might mediate the tumor-suppressing effect by facilitating the action of Th1 cells in stimulating and activating cytotoxic T cell immune responses targeting the tumor (113, 156). Another putative mechanism is manifestation of memory stem cell-like properties in cells that differentiate into Th1/Th17 cells and produce IFN-γ (113, 157). On the contrary, transcriptomic data analysis has revealed a strong correlation between Th17 cells and angiogenesis metagenes (158). This suggests that the robust infiltration of Th17 cells into a tumor site possesses the ability to induce VEGF-A secretion, which, in turn, promotes neoangiogenesis and places patients with GBM in unfavorable scenario. Another study has suggested that the inefficiency of immunotherapy in GBM patients may be attributed to the prevalence of Th17 lineage in the CD4^+^ T cell phenotype (159). This prevalence creates a hostile environment for the infiltration of cytotoxic T lymphocytes, potentially through CD39-mediated adenosine production and/or the recruitment of myeloid cells.

Of interest, several studies have provided evidence of the decisive role of T17 cells in the context of anti-cancer strategies based on the immunogenic cell death approach (ICD, **Box 2**). The use of chemotherapeutic drugs that induce ICD can attract γδT17 cells in the TME, thereby facilitating subsequent cognate anticancer T cell responses (160). Building on this initial work, it has been shown that a dendritic cell (DC) vaccine, based on whole glioma GL261 cell lysates pulsed with ICD-induced photodynamic therapy, has an activated Th17 signature characterized by an increased expression level of Tgfb-3, IL-6, and IL-23a genes (114) (**Figure 1**). This approach demonstrates prophylactic and therapeutic efficiency in a murine orthotopic glioma model (114). Importantly, blocking the transcription factor responsible for the Th17 response (e.g., RORγt) transforms the TME by depleting IL17 within the tumor which subsequently leads to a significant decrease in the prophylactic efficacy of the dendritic cell (DC) vaccine (114). The design of DC vaccines, based on another ICD-inducer for glioma cell death, leads to an increase in tumoral accumulation of Th1/CTL/Th17 cells in both prophylactic and therapeutic regimens *in vivo*. This approach is associated with prolonged overall survival in patients with newly diagnosed GBM, as indicated by the conducted meta-analysis (115) (**Figure 1**).

In conclusion, a solution for the immunologically ‘cold’ TME roadblock and a mechanism transforming the TME into an immune-favorable TME will pave the way for anti-glioma immunotherapy. Given the diverse nature of the TME, one potential approach could involve evaluating its unique composition for each individual patient. However, this remains costly and technically challenging. The application of ICD (**Box 2**) in the immunotherapeutic approaches against gliomas holds great promise for overcoming T-cell dysfunction in the TME, leading to the activation of specific adaptive immune responses and enhancing ongoing therapy.

## 4 Which immune profiles are specific to gliomas?

**Figure 2:**
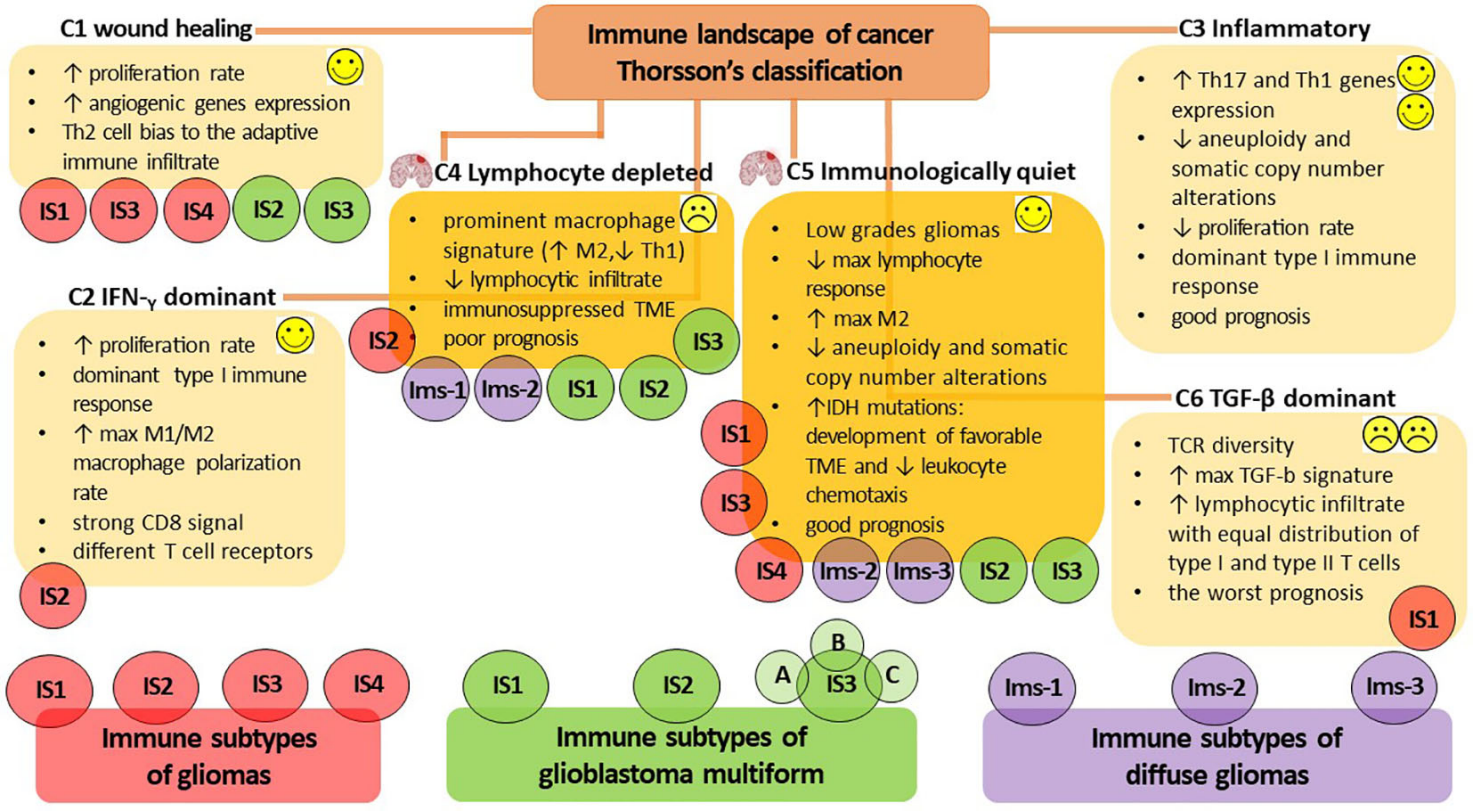
The immune subtypes characteristic of gliomas. The classification of V. Thorsson et al. defines six major immune subtypes (C1-C6), which have a specific pattern of immune responses determining the prognosis for patients (161). The most characteristic immune subtypes of gliomas are C4 (lymphocyte depleted), associated with poor outcome to immunotherapy, and C5 (immunologically quiet), indicating a favorable prognosis. Bioinformatics analysis by S. Ma et al. identified four immune subtypes of gliomas (IS1-IS4) that differ in diverse molecular, cellular, and clinical presentation (15). The IS1, IS3, and IS4 phenotypes predominantly overlapped with the C1 (wound healing) and C5 (immunologically quiet) immune subtypes of Thorsson’s classification, whereas IS2 overlapped with the C2 (IFN-γ dominant) and C4 (lymphocyte depleted) immune profiles. The additional presence of a high proportion of C6 (TGF-β dominant) in the IS1 subtype was consistent with the worst prognosis. Omix data analysis performed by Q. Zhou et al. allows the definition of three immune subtypes (Ims1-3) associated with the prognosis of diffuse glioma (162). Ims1 overlapped the C4 immune profile of Thorsson’s classification, the Ims2 subtype was mainly enriched in C4 and C5, whereas the most favorable prognosis was accompanied by Ims3 immune profile mainly enriched in C5 of Thorsson’s immune subtype. H. Lin et al. classify three immune subtypes of glioblastoma multiforme (IS1-3) (163), whereas IS1-3 mainly overlap the C4 immune subtype of Thorsson’s classification; however, the IS2 и IS3 subtypes can also overlap the С1 and C5 immune profiles. The proposed bioinformatic classification method subdivides the IS3 subtype into the next three subtypes (IS3A-C), of which IS3A is characterized by a more inflamed tumor microenvironment and showed significantly better patient outcome. Thus, the assessment of the rationale for anti-tumor vaccination and its efficacy in patients diagnosed with glioma is currently challenged due to the lack of a unified classification for highly accurate immune profiling of patients. Success in developing a patient-specific immune profile approach requires the development of advanced methods for constructing highly sensitive predictive bioinformatic models.

Given the remarkable heterogeneity of glioma and its TME, a more precise identification and characterization of the immune profile would be highly valuable for predicting glioma progression. Additionally, it would aid in predicting patient prognosis, facilitating the selection of personalized treatment regimens and determining the patient’s response to immunotherapy. To date, there is no unified classification for highly accurate determination of the immune profile of a patient diagnosed with glioma to predict the rationale for anti-tumor vaccination and its efficacy. However, in 2018, Thorsson et al. published the results of a comprehensive immunogenomics analysis of more than 10,000 tumors across 33 diverse cancer types in data compiled by TCGA (https://www.cancer.gov/tcga). This analysis identified six major immune subtypes (C1-C6) (**Figure 2**), which have characteristic patterns of immune responses to predict the prognosis for patients (161). Among the identified immune subtypes, the С4 (lymphocyte depleted) and C5 (immunologically quiet) immune subtypes were the most characteristic for gliomas. The С4 immune subtype is characterized by the prominent macrophage signature, with Th1 suppressed and a high M2 macrophages response, and low lymphocytic infiltrate. Such an immune profile is consistent with an immunosuppressed TME, for which a poor outcome would be expected. The C5 immune subtype is mainly characteristic of low-grade gliomas, the immune profile of which consists of the lowest lymphocyte and highest macrophage responses with high M2 macrophage content. Lower levels of aneuploidy and overall somatic copy number alterations are also typical of С5. High prevalence of IDH mutations (**Box 3**) suggests their participation in the formation of a TME composition favorable for the outcome (172) and decreased leukocyte chemotaxis, leading to fewer tumor-associated immune cells and better treatment outcome (173).

Box 3 **Tumor antigens and immunotherapy: a brief overview.**

Tumor antigens are traditionally divided into tumor-associated antigens (TAAs) and tumor-specific antigens (TSAs). Several studies suggest that mutations of isocitrate dehydrogenases (IDH1/2), which represent one of the main characteristics of glioma cells and occur at specific codons in IDH1 and IDH2, are attractive candidates for glioma immunotherapy (164, 165). Among TSAs, neoantigens constitute a group of antigens of viral origin and antigens resulting from mutations in cellular proteins and can be divided into shared and personalized neoantigens. The shared neoantigens are widespread tumor mutations in specific domains of p53, KRAS, NRAS, and other oncogenes. These mutations are found in malignant tumors originating from cells of a wide variety of tissues.

Personalized neoantigens are tumor antigens derived from specific tumor mutations that are unique to a given patient. Because personalized neoantigens differ in patients with the same tumor type, the development of approaches for their identification has received particular attention (166–168). This focus is largely due to the low efficacy of ongoing immunotherapeutic strategies against gliomas associated with the brain’s immune-privilege, the presence of the BBB, high tumor antigenic heterogeneity i.e., the presence of multiple antigen epitopes), and the formation of an immunosuppressive tumor microenvironment (169). In this regard, personalized therapeutic vaccination with the patient’s own tumor tissue based on the identified personalized neoantigens can potentially increase the effectiveness of specific antitumor immunity and represents a fundamentally new therapeutic option for patients with glioma (170, 171). Thus, an ideal strategy in the design of anti-cancer vaccines is to target self-antigens that combine multiple epitopes. The development of such a strategy is inextricably linked to the bioinformatics analysis of publicly available databases of cancer patients and/or experimental animals, and it is expected that targeting personalized neoantigens will be the primary therapeutic option.

On the other hand, considerable expansion of the panel of *in silico* studies has made Thorsson’s classification of gliomas more ambiguous. Bioinformatic immune-associated gene clustering data from TCGA and Chinese Glioma Genome Atlas cohort data (CGGA, http://www.cgga.org.cn/) identified four immune subtypes (IS1-IS4) of gliomas (**Figure 2**) differing in diverse molecular, cellular and clinical presentation (15). The immune “active” phenotypes IS1 and IS4 are associated with shorter survival and characterized by elevated tumor mutational burden or somatic mutation rates (total numbers of mutations, SNPs and SNVs) and high expression levels of immune checkpoints. This pattern suggests the presence of an immunosuppressive TME that could interfere with the activation of immune responses and the efficacy of ongoing vaccination. On the other hand, the immune “suppressive” phenotypes IS2 and IS3 had lower levels of tumor immune infiltration and elevated expression of ICD regulators, and it was accompanied by good prognosis for patients and their responsiveness to vaccination (15). At the same time, the IS1, IS3 and IS4 phenotypes predominantly overlapped with C1 (wound healing) and C5 (immunologically quiet) immune subtypes of Thorsson’s classification, whereas IS2 overlapped with C2 (IFN-γ dominant) and C4 (lymphocyte depleted) immune profiles. In the IS1 subtype, the additional presence of a large proportion of C6 (TGF-β dominant) was in line with the worst prognosis for patients (15, 161).

Another computational omics data analysis of the TCGA and CGGA cohorts defines three immune subtypes (Ims1-3) associated with the prognosis of diffuse glioma (**Figure 2**) (162). The Ims1 subtype has main similarities with the C4 immune profile of Thorsson’s classification and is associated with the shortest median survival of patients (14 months). The authors assumed that patients with the Ims1 subtype are more suitable for RNA-based vaccination, as it was characterized by high levels of tumor mutational burden, suggesting that tumor mutations might generate immunogenic neoantigens and a high expression level of immune checkpoints (e.g., PD-1, CD40, PD-L1, CD80 and CD86) and ICD modulators (e.g., FPR1, CXCL10, ANXA1, and MET) (174, 175). The subtype Ims2 was mainly enriched in C4 and C5 of Thorsson’s immune subtypes, whereas the most favorable prognosis (median survival time of 94.5 months) was accompanied by an Ims3 immune profile mainly enriched in C5 immune subtypes (162).

Machine learning-based data analysis of TCGA and CGGA cohorts based on the expression of integrated immune-related gene profiles divides patients with GBM into three immune subtype (IS1-3) and provides a guideline for identification of tumor antigens intended for the design of anti-GBM mRNA vaccines (**Figure 2**) (163). IS1-3 mainly overlap C4 of Thorsson’s immune profile and is less associated with the C1 and C5 immune profiles. GBM having IS1 (intermediate state) and IS2 (immunologically “hot”) lack regulatory immune cells and immunosuppressive antigen-presenting cells, resulting in T cell activation and the potential for inducing a stronger immune response to ongoing vaccination. IS3 was classified as an immune-cold subtype with the worst prognosis for patients and was characterized by highly complex and problematic TME. Interestingly, the proposed bioinformatics approach allows the identification of intra-cluster heterogeneity in GBMs and subdivides the IS3 subtype into the next three subtypes (IS3A-C), among which IS3A showed significantly better survival than the other two subtypes. IS3A is characterized by a more inflamed microenvironment with more activated B cells, cytotoxic T cells and NK cells.

It is important to develop approaches for precise determination of patient-specific immune profiles and evaluation of the effectiveness of immunotherapeutic strategies against gliomas. Such developments would depend on the development of artificial intelligence and machine learning methods for multi-omics data analysis, which can help to build highly sensitive predictive models. Such models would make it possible to search for specific neoantigens (**Box 3**) that could form the basis of personalized immunotherapy, including vaccination strategies. However, the bioinformatics predictive models must be confirmed in clinical trials. It is interesting that one advantage of whole-tumour vaccine approaches is that they do not depend on specific haplotypes. Such approaches would allow the immune system to target a wide range of tumor antigens, including any neoantigens that may be presented. This reduces the likelihood of emergence of immune escape variants.

## 5 Identification of glioma antigans and neoantigens for vaccination

The main goal of the existing vaccination strategies is to identify and target tumor antigens. Identification of mutated and aberrantly expressed intrinsic tumor antigens has historically been a laborious task. Mutant antigens are usually expressed in a tumor-specific manner, and aberrantly expressed antigens are often common in different cancer cell types. Therefore, they are more likely to be the focus of therapeutic anticancer vaccines (**Box 3**). In 2008, tumor cells genome sequencing revealed that most cancers have somatic mutations (176, 177). In 2012, two independent studies by the groups of Uğur Sahin (178) and Robert Schreiber (179), based on next-generation sequencing and *in silico* epitope prediction in combination with advanced immunological analysis, proposed a highly accurate algorithm to identify different neoantigens in tumor cells. This approach reduces the time for identifying targeted neoantigens to a few weeks, as opposed to months for more routine antigen cloning approaches. In 2016, Gavin Dunn’s group identified potential targets for generating anti-tumor CD8 T-cell responses in a mouse model of glioblastoma (180). Expressed tumor-specific mutations were characterized by whole exome DNA and RNA sequencing of GL261 cells. Several *in silico* algorithms for the prediction of MHC I binding were then applied to identify a putative high-affinity H-2Db-restricted neoepitope, and then the immunogenicities of the predicted neoantigens were evaluated. Using IFN-γ Enzyme-Linked Immunospot (ELISPOT) and tetramer assays, the authors confirmed the presence of an endogenous CD8 T-cell response specific for H-2D^b^-restricted Imp3D_81N_ neoantigens. In addition, endogenous T-cell populations neoantigen-specific to Imp3D_81N_ and Odc1Q_129L_ in the brain and lymph nodes were detected. This is one of the first studies in which a neoantigen discovery pipeline for the identification of potential therapeutic target candidates was established, providing proof of principle for further analysis of the mechanisms of action of T cell-activating immunotherapeutic approaches in preclinical models of glioblastoma.

More recently, a clinical trial tested personalized neoantigen-targeting vaccines by immunizing patients with newly diagnosed glioblastoma after surgical resection and conventional radiotherapy (phase I/Ib, NCT02287428 ClinicalTrials.gov). The assessment of whole-exome sequencing and RNA-seq data generated from the tumor tissue obtained at the time of diagnostic resection allowed the prediction of personal neoantigens (**Box 3**) based on analysis of binding affinity to individual HLA alleles. As a result, the personalized vaccine consisted of up to 20 specific peptides of 20–30 amino acids, which generated circulating polyfunctional neoantigen-specific CD4^+^ and CD8^+^ T cell responses enriched in a memory phenotype, along with an increase in intratumoral T cell infiltration (181). Moreover, by using single-cell T cell receptor analysis, the authors demonstrated that neoantigen-specific T cells from the peripheral blood migrate into an intracranial glioblastoma tumor. This indicates that neoantigen-targeting vaccines could favorably change the immune milieu of glioblastoma.

Extensive bioinformatics analysis is currently being applied as a valuable tool to search for glioma-specific neoantigens in patient biomaterial. In a recent study, four antigens, namely ANXA5, FKBP10, MSN and PYGL have been identified as associated with a favorable prognosis for patient and have been proposed as potential target antigens for mRNA vaccine production against glioma (14). Additionally, another four potential antigens including TP53, IDH1, C3 and TCF12, have been identified as significant contributors to glioma growth intensity and have the capacity for direct procession and presentation to CD8 T cells when there is sufficient lymphocyte infiltration to induce an immune response (15). The overexpression of three mutated tumor antigens, KDR, COL1A2, and SAMD9 is associated with the activation of professional antigen-presenting cells (e.g., positive correlation with abundance of B cells, macrophages and DCs) and an unfavorable prognosis for patients with diffuse glioma (162). The authors have shown promising prospects for patients with the Ims-1 immune subtype (**Figure 2**) for mRNA vaccination targeting these three mentioned antigens (162). Lin et al. have identified six overexpressed and mutated tumor-associated antigens including ARHGAP9, ARHGAP30, CLEC7A, MAN2B1, ARPC1B and PLB1, which are associated with poorer GBM prognosis and increased infiltration of antigen-presenting cells (i.e., B cells, macrophages, and DCs), making them suitable targets for vaccine use (163). It is essential to keep in mind that although some novel glioma antigens have been identified, their therapeutic relevance requires validation in relevant mouse models and in the clinical trial. A recent clinical trial of a peptide vaccine targeting a mutation in codon 132, which encodes the IDH1 gene (IDH1(R132H)), containing a common clonal neoepitope presented on MHC II, has shown promising results, with a three-year survival benefit for patients with astrocytomas (WHO grades 3 and 4, NCT02454634 ClinicalTrials.gov) (182).

At this point, it remains clear that there are several challenges and timeframes associated with the clinical translation of personalized neoantigen-based vaccines. The manufacture of individualized vaccines is a complex and time-consuming process, and there is uncertainty in selecting the optimal platform for neoantigen detection, as well as a lack in consensus regarding the most suitable vaccine delivery platform (160). Moreover, immunoinformatic methods for identifying neoepitopes in cancer genomes are diverse and have not been well-validated (183). Therefore, the clinical development landscape of personalized neoantigen-based vaccines is still evolving. As more clinical trials are conducted, the efficacy and safety of personalized neoantigen-based vaccines will become clearer, and the timeframes for their clinical translation may become more defined. Thus, there is a need to expand our knowledge of glioma antigens and neoantigens, as well as the methods for their identification. Many more interesting and challenging findings are expected, along with potentially applicable therapeutic approaches in the field.

## 6 Concluding remarks and future perspectives

Immunotherapy is currently considered an integral part of complex therapeutic procedures designed to increase the curability of a wide range of cancers. The involvement of immune cells and activation of a T-cell immune response allows the effective eradication of tumor cells that remain viable after conventional therapy, eliminating the risk of secondary tumor development and metastasis. Several immunotherapeutic strategies are currently used and adapted for the anti-cancer treatment (e.g., immune checkpoint inhibitors, chimeric antigen receptor (CAR) T cell therapy, oncolytic virus-based therapy, gene-mediated cytotoxic immunotherapy, and therapeutic anti-tumor vaccination). But approaches based on the induction of ICD (**Box 2**) draw a lot of attention, as they can act as a powerful tool to multiply immunotherapeutic efficacy. However, despite great promise and abundant evidence supporting the necessity for immunotherapy, its application in glioma treatment has yet to achieve major breakthroughs in the clinic. This is due to a number of challenges that need to be addressed to achieve enhanced immunotherapy against gliomas.

The main challenge in glioma immunotherapy is the high hostility of glioma TME to T-cell infiltration and activation. Gliomas, particularly high-grade gliomas like glioblastoma multiforme, exhibit an immunologically “cold tumor” phenotype characterized by high TME heterogeneity, as well as tumor cell-intrinsic mutations and tumor-extrinsic mechanisms (106) (**Figure 1**). Recent data have highlighted the significant potential of inducing ICD in glioma, which leads to the recruitment of Th17 cells to the TME, thereby transforming it into an immunologically “hot” environment. This activation of antitumor immunity fosters an effective response to ongoing immunotherapy (114, 115). Therefore, it is reasonable to anticipate that the ICD approach will pave the way for improved immunotherapy against glioma.

During the analysis of the immune profiles of patients with gliomas (**Figure 2**), it becomes evident that the significant heterogeneity of gliomas and their TME excludes the possibility of a unified classification of a patient’s immunological profile. Development of a unified classification will be helpful for accurate assessment of tumor progression and responsiveness to therapy. Strictly speaking, it seems that a personalized immunological profile needs to be defined for each glioma patient. In this regard, different artificial intelligence and machine learning methods for multi-omics data analysis are being actively developed to build highly sensitive predictive models that can search for specific neoantigens (**Box 3**) for personalized immunotherapy, including vaccination strategies. However, it will take some time to verify them *in vivo* and even more so in clinical trials. Nevertheless, looking back at the currently accepted Thorsson’s classification (**Figure 2**), it can already be noted that increased expression of Th17 and Th1 genes is one of the essential components for best prognosis.

Due to the high heterogeneity of gliomas, there is significant interest in exploring novel approaches to identify neoantigens derived from specific tumor mutations unique to each patient (166–168). Personalized therapeutic vaccination, utilizing the patient’s own tumor tissue based on these identified personalized neoantigens, represents a fundamentally new therapeutic option for glioma patients and could increase the effectiveness of specific antitumor immunity (170, 171). Remarkably, in the design of vaccines against glioma, it is possible to combine multiple mRNA sequences of patient-specific neoantigens with mRNA sequences of adjuvants, potentially boosting the antitumor immune response. A well-known technology, TriMix, includes mRNA encoding costimulatory molecules such as CD70 and CD40 ligand (CD40L), and active TLR4, and it is being enhanced to facilitate effective activation of DC cross-presentation and priming of CD8^+^ T cell responses (184–187). Alternatively, mRNA sequences of cytokine cocktail (e.g., IL-23, IL-36γ) and the T cell costimulatory OX40L, both actively involved in combating the immunosuppressive TME and promoting durable anticancer immunity (188), can be employed as independent adjuvants or in combination with the TriMix technology. Further development of this approach in glioma immunotherapy will open new avenues for the creation of more effective therapeutic regimens. These regiments will be tailored to analyze individual rare immunogenic mutations, leading to the most personalized treatment strategies.

## Author contributions

TM: Conceptualization, Data curation, Formal analysis, Funding acquisition, Investigation, Writing – original draft, Writing – review & editing. VT: Data curation, Formal analysis, Investigation, Writing – original draft. EG: Formal analysis, Investigation, Writing – original draft, Data curation. OK: Data curation, Formal analysis, Writing – review & editing. MV: Conceptualization, Supervision, Writing – review & editing. DK: Conceptualization, Supervision, Writing – review & editing.
